# FuFiHLA: a tool for full-field HLA typing from long-read data

**DOI:** 10.1093/bioinformatics/btag231

**Published:** 2026-05-05

**Authors:** Jingqing Hu, Qian Qin, Heng Li, Ying Zhou

**Affiliations:** Department of Data Science, Dana-Farber Cancer Institute, Boston, MA 02115, United States; Department of Data Science, Dana-Farber Cancer Institute, Boston, MA 02115, United States; Department of Biomedical Informatics, Harvard Medical School, Boston, MA 02115, United States; Division of Rheumatology, Inflammation, and Immunity, Brigham and Women’s Hospital, Boston, MA 02115, United States; Department of Data Science, Dana-Farber Cancer Institute, Boston, MA 02115, United States; Department of Biomedical Informatics, Harvard Medical School, Boston, MA 02115, United States; Medical and Population Genetics Program, Broad Institute of MIT and Harvard, Cambridge, MA 02142, United States; Department of Data Science, Dana-Farber Cancer Institute, Boston, MA 02115, United States

## Abstract

**Motivation:**

Allele typing for Human Leukocyte Antigen (HLA) genes has many important clinical applications. Popular short-read typing can only accurately distinguish alleles at the coding sequence level, which potentially limit our understanding of the effect of variants in non-coding region. Long read data has been proved to be useful in typing HLA alleles in full resolution, but only a few tools are publicly available and with significant limitations in practical application.

**Results:**

We developed FuFiHLA, a lightweight open-source software, to type HLA alleles. Currently it supports typing alleles of six HLA genes (HLA-A, HLA-B, HLA-C, HLA-DRB1, HLA-DQA1, and HLA-DQB1) from long reads. Evaluation using 233 PacBio HiFi WGS samples from HPRC shows that FuFiHLA achieves 99.6% accuracy in the full field allele typing and QV as 51.8 for consensus allele sequence construction. Additional testing on four Nanopore R10 reads demonstrates slightly reduced accuracy in the fourth field.

**Availability:**

FuFiHLA is available at https://github.com/jingqing-hu/FuFiHLA under MIT License.

## 1 Introduction

The Human Leukocyte Antigen (HLA) region is located on the short arm of chromosome 6, specifically on band 6p21.3, and covers more than one hundred coding genes that play crucial roles in human immune system. This region has been implicated in over hundreds of diseases (Shiina et al. 2009). Due to the need of recognizing unpredicted antigens, several HLA genes exhibit exceptionally high within-species diversity even within the coding region. For instance, pairwise differences are 3% for HLA-A, 4% for HLA-DRB1, and 5% for HLA-DQB1 (Zhou et al. 2024), whereas the genome-wide average is 0.1% in human (The 1000 Genomes Project Consortium et al. 2015) and about 1.23% between human and chimpanzee (Suntsova and Buzdin 2020).

Regarding the high polymorphism in HLA genes, a nomenclature system has been developed for distinguishing gene sequences or alleles (Marsh et al. 2010). A specific gene sequence, or an allele, is assigned a name consisting of four fields, addressing the difference in antigen binding affinity (field 1), in peptide (field 2), in coding nucleotide (field 3), and in intron nucleotide (field 4). An extra suffix tag may be appended to denote expression changes.

HLA typing is to infer the HLA allele name from biospecimen based on a pre-exist reference database. In this work, we will focus on using PacBio HiFi reads of DNA sequences (Lang et al. 2020), and extending to Nanopore R10 data with a few modifications in parameter setting. The IPD-IMGT/HLA is used as the standard reference data set for HLA allele typing (Barker et al. 2023). In the version 3.59, it consists of 41003 distinct alleles covering 47 HLA genes.

With a known reference allele data set, there are two major computational strategies for HLA allele typing from DNA sequences: one is to infer the best allele combinations from reference database to explain the observed reads, the other is to recover or reassemble the allele sequence and type the allele by comparing to the reference data set (Szolek et al. 2014, Dilthey et al. 2019, Klasberg et al. 2019, Claeys et al. 2023, Song et al. 2023). Using short read data, the first strategy is less robust with undocumented alleles, while the second strategy might be affected by assembly errors. Thus, both strategies can hardly achieve high accuracy in full-field HLA typing (Thuesen et al. 2022, Dashti et al. 2024).

Long read sequencing has been proved to be useful for typing full field HLA due to the long extension of read length. For example, PacBio SMRT sequencing was proved to be useful in constructing full length sequences of HLA class I genes and class II genes (Mayor et al. 2015, 2019, Turner et al. 2018, Creary et al. 2019, French et al. 2024). There are three recently published typing tools taking long read data but with limitations: HLA*LA (Dilthey et al. 2019) only outputs the first three fields nomenclature; StarPhase (Holt et al. 2024) (a new version of HiFiHLA) only supports PacBio HiFi data; SpecImmune (Wang et al. 2026) needs an aligned bam with a specific version of reference genome, which is less convenient in practice. In this work, we will present a new method for HLA allele typing on the six graft transplant genes (HLA-A, HLA-B, HLA-C, HLA-DQA1, HLA-DQB1, and HLA-DRB1), it can take both aligned and raw/unaligned long reads as input, typing HLA allele in full-field and constructing consensus allele sequences. FuFiHLA is developed for and evaluated on PacBio HiFi and Nanopore R10 whole genome sequencing data.

## 2 Methods

**Figure 1:**
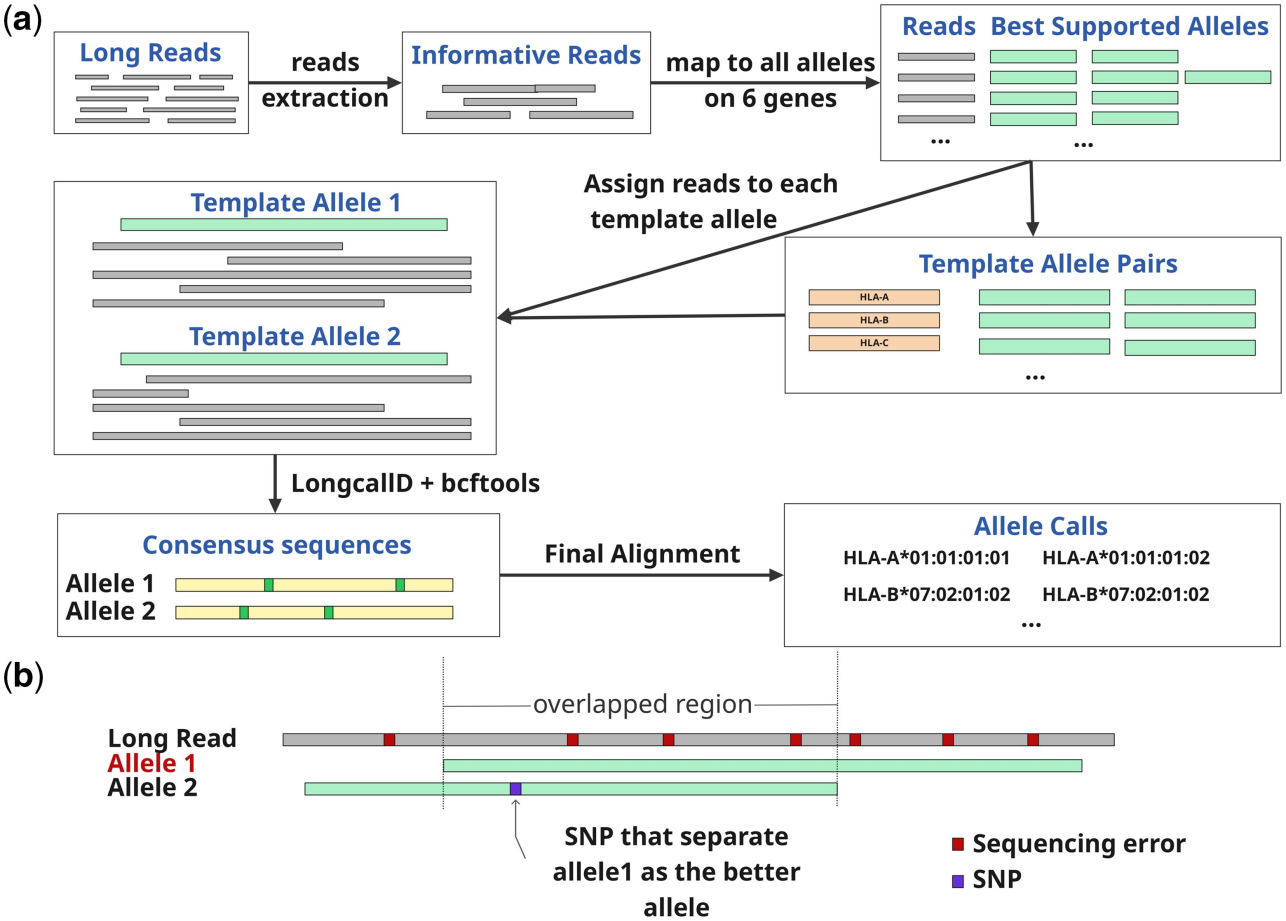
Overview of FuFiHLA. (a) Informative reads are extracted from raw reads by mapping them to the anchor alleles (Section 2.1) and further mapped to all alleles from targeted genes to find out template allele pair for each gene (Section 2.3). Then those informative reads are assigned to each of the template allele for variants calling and consensus gene sequence construction (Section 2.3). Reconstructed sequences that include variants from template allele are further mapped to all alleles of targeted genes for allele typing (Section 2.4). (b) MOI method for choosing better alignment (Section 2.2).

This pipeline processes reads through sequential steps to output allele sequences and allele types (Fig. 1a).

### 2.1 Informative read extraction

We used a subset of IPD-IMGT/HLA alleles as anchors to extract informative reads from raw reads. When bam and the gene annotation of the reference are available, we can also use reads overlapped with the targeted genes as input. In our evaluation, both generated nearly identical allele typing results, but with slightly difference in consensus sequence construction.

To construct anchor allele set, we clustered all IPD-IMGT/HLA allele sequences with cd-hit (identity cutoff = 0.95) (Li and Godzik 2006), and from each cluster we selected one allele as anchor allele to represent that allele cluster. We also added allele sequences to the anchor set to filter reads from paralogous genes. In total there are 136 anchor alleles selected, covering the six targeted genes and 37 other HLA genes.

The reads were mapped to all anchor alleles and further extracted as informative reads based on the overlapping with anchor alleles from the six targeted genes. Particularly, an informative read should be covering at least 40% length of any targeted anchor allele or covering at least 200 bps from one end of a targeted anchor allele. Due to the similarity among HLA paralogous genes, one read can be mapped to several alleles even from different genes which may introduce additional noise. Thus, the allele-to-read mappings were removed if the mismatch rate is 10 times higher than the lowest mismatch rate of the same read.

### 2.2 Alignment comparison

To determine the better alignment between two alleles to a read, we restrict the comparison of **M**ismatch in the **O**verlapped mapping **I**nterval (**MOI**) (Fig. 1b). This design is tested to be more practical than using mismatch rate directly due to the existence of sequencing error and the nature of allele length variation. With the MOI method, the better alignment includes a smaller number of differences in the overlapped mapping interval. A difference could be a mismatch or a contiguous gap. Gaps of <3 bp in size are ignored as they are prone to sequencing errors. For reads with higher error rates, such as the Nanopore R10 data, more ‘tie’ situations are created in the comparison by ignoring sequence difference with at most one indel or substitution event.

### 2.3 Template allele pair selection and consensus allele sequence construction

A pair of template alleles for each targeted gene are used as references to construct consensus allele sequences from informative reads, based on the assumption that the target sample has exact two copies of each of the six genes. Template allele pair is a combination of reference alleles of the highest agreement with the reads. In total 19 782 allele sequences of the targeted genes are used in the selection.

To select a pair of template alleles for each of the targeted genes in one sample, we constructed a pool of alleles (*P*_allele_) to iterate allele pairs and a pool of reads (*P*_read_) that belongs to the specific gene. When multiple alleles are mapped to the same region of a particular read, the best-match allele(s) are selected by the MOI method through pairwise comparison. The selected allele(s) are added to the allele pool *P*_allele_, and the supporting read is added to the read pool *P*_read_. The mappings between selected alleles and their supporting read are also recorded.

For an allele A, a three-metric tuple S(A) = (*n*, *c*, *m*) is calculated, where *n* is the total number of unique supporting reads, *c* is total read coverage, and *m* is total number of matched base pairs. If one read supports several alleles, then its contribution to *c* will be divided by the total number of supported alleles.

The three-metric tuple for a pair of alleles, noted A and B, is calculated as S(A,B)=S(A)+S(B). We rank allele pairs based on the three-metric tuple S(A,B) in decreasing order and the allele pair of the first rank is selected as the template allele pair. Practically, we found that top 15 alleles for HiFi reads and top 30 alleles for R10 reads with highest coverage was good enough to select the proper template allele pair.

Once the template allele pair is selected, reads are assigned/phased to each of them through MOI method. If the mappings to each template allele are equal, then the read is assigned to both alleles. Since it tends to give two different alleles even though for the homozygous sites, we added an additional filtration to the template alleles that if one allele’s mapping coverage is four times smaller than the other, the template allele with less coverage will be removed and the site is forced to be homozygous.

With phased reads and the template allele, we applied longcallD (Gao et al. 2026) for variants calling, which performs multi-sequence alignment between reads and is more robust to inconsistent alignment around indels. We used “bcftools consensus” (Danecek et al. 2021) for allele sequence construction. Only major variants supported by at least three reads are used for variants calling and consensus sequence construction.

### 2.4 HLA Allele typing

HLA allele typing is based on a penalty score defined by the mismatch between consensus allele sequence and reference allele sequence. The penalty is set as 9999 for each exonic mismatch and 1 for each intronic mismatch. The allele with the lowest penalty score is selected as the typing allele. Partial alleles are only applied when the target consensus sequence does not have a perfect gene sequence match but with a perfect coding sequence match. A novel allele will be typed as the closest allele with full gene length in the IPD-IMGT/HLA database, with a cigar string (Li 2018) indicating the nucleotides difference. Additionally, consensus sequences of typed alleles are also provided.

## 3 Results

We first evaluated the performance of our HLA typing tool on 233 HiFi samples from the Human Pangenome Reference Consortium (Supplementary Figs. S1-2), which includes 45 Americans, 69 Africans, 37 South Asians, 51 East Asians and 31 Europeans. The average read depth is 41X and average read length was about 15 kb. The error rate of HiFi long reads is <1% (Liao et al. 2023).

Regarding the assembly-based annotation as ground truth (Zhou et al. 2024), we compared FuFiHLA with three other long read typing tools, HLA*LA(1.0.4), StarPhase(1.3.2) and SpecImmune(1.0.0), across the six targeted genes at different resolutions. In our evaluation, both StarPhase and FuFiHLA demonstrated high typing accuracy, and FuFiHLA has even higher typing accuracy than StarPhase at all resolution (Table 1, Supplementary Table S1).

**Table 1:** Accuracy of long read HLA typing on six targeted genes across 233 HPRC HiFi samples.

Software	1-field (%)	2-field (%)	3-field (%)	4-field (%)	QV
StarPhase	99.89	99.86	98.92	97.66	45.8
FuFiHLA (raw)	100	99.89	99.89	99.58	51.8
FuFiHLA (bam)	100	99.93	99.93	99.58	52.6

* For FuFiHLA typing from raw reads, allele to allele comparison can be found in Supplementary Table S2, and per gene accuracy can be found in Supplementary Table S3. Immuannot annotation is taken as the ground truth. Immuannot may report a “new” field for a novel allele such as “HLA-A*24:02:01: new”. In this case, accuracy is only evaluated on the first three fields. In contrast, a truth allele having <4 fields in the database, such as “HLA-A*68:17:01”, is still used for full-field evaluation. QV scores are calculated by comparing consensus allele sequences to the de-novo assemblies.

We also evaluate the base pair consistency of gene sequence construction by aligning the constructed gene sequences to the de novo assemblies of each of the 233 samples from HPRC. Among the 2796 allele sequences, FuFiHLA (raw/unaligned read as input) gave 2752 (98.42%) perfectly matches. Among the 44 gene sequences with mismatched nucleotides, 36 of them are differed by only indels in homopolymer or short tandem repeats region (Supplementary Table S4). Using bam for reads extraction has similar performance (Supplementary Table S5). Meanwhile StarPhase provided 2640 (94.42%) perfect matches and 156 mismatches (Supplementary Table S6). The overall QV score of consensus allele sequence is 51.8 for FuFiHLA and 45.8 for StarPhase (one wrong call was excluded).

Using Nanopore R10 reads would lead to reduced accuracy for FuFiHLA in the fourth field. Among four testing samples [HG002 from GIAB (Talenti 2023) and three non-cancer samples from CASTLE panel (Park et al. 2024)], it achieved 100% accuracy for the first three fields but dropped to 87.0% for the forth field, mainly due to the increasing sequencing error of homopolymers in introns (Supplementary Table S7).

## 4 Discussion

In this work we presented a new method, FuFiHLA, which can accurately type six HLA genes from WGS long reads. Based on the evaluation on 233 HPRC HiFi samples, FuFiHLA achieved 99.58% accuracy at full field allele typing. StarPhase is another method developed recently by PacBio, which is less accurate in both allele typing and consensus allele sequences construction. StarPhase is faster than FuFiHLA when taking bam (aligned) as input, but slower when accounting for bam preparing time because using anchor sets is more efficient in CPU usage and memory occupation than constructing bam to extract informative reads (Table 2, Supplementary Table S8).

**Table 2:** Runtime in minutes.

	Sample	a	b	c	d	e
bam_size	(GB)	33	78	98	111	134
Alignment (minute)	FuFiHLA (raw)	270	772	941	919	1060
	StarPhase	1759	4173	5049	5674	6899
	SpecImmune	1883	4331	5289	5874	6901
	HLA*LA	1759	4173	5049	5674	6899
Typing (minute)	FuFiHLA (raw)	32	63	76	94	126
	FuFiHLA (bam)	21	41	46	50	53
	StarPhase	3	4	3	3	4
	SpecImmune	33	36	41	37	40
	HLA*LA	255	714	682	588	856

Alignment time for StarPhase is the time used to construct bam file. Sample name for each column: (a) HG002; (b) HG00733; (c) NA19240; (d) HG00621; (e) HG02622.

Taking advantage of the large number of reference HLA gene sequences in IPD-IMGT/HLA, we are able to select a template pair that minimizes the difference between reference alleles and targeted alleles, which makes it more robust in allele typing and allele sequence reconstruction, even when the read depth is low. For example, HG002 has 10-fold coverage and could still correctly type all 12 alleles with only a few intronic consensus errors due to indels in homopolymer and short tandem repeats, which is the dominant error mode of FuFiHLA in general (Supplementary Table S9).

While FuFiHLA uses known full-length gene sequences as templates to seed read alignment, it always reconstructs complete sequences for all alleles. It thus can report if an allele is novel and whether it leads to unknown coding changes. Users also have the option to store the consensus sequences and retype them with future versions of the IPD-IMGT/HLA database at minor computing cost.

It is worth acknowledging that long-read targeted sequencing is a well-established approach for HLA typing in clinical settings offering lower costs, faster turnaround times, and higher coverage across specific HLA loci (Suzuki et al. 2018, Matern et al. 2020, Pedersen et al. 2025). However, specialized bioinformatic tools, which are mostly commercial or proprietary, often lose their efficacy when applied to lower coverage whole genome sequencing (WGS) reads. This WGS type data is more common in large population study cohorts, such as the *All of Us* and Singapore pangenome projects (All of Us Research Program Investigators et al. 2019, Sarashetti et al. 2024). In our evaluation, we excluded those tools due to limited public availability.

While FuFiHLA is designed for long-read data and can, in principle, be applied to targeted sequencing, doing so carries risks of reducing accuracy due to platform bias and errors such as allele dropout, allelic imbalance, and PCR chimeras (Laver et al. 2016, Osoegawa et al. 2019). We did not include targeted sequencing data in our evaluation because we could not source high-quality datasets with verified ground truth. We hope to collaborate with clinical labs in the future to extend our work. Furthermore, while FuFiHLA currently types six classical HLA genes, we plan to extend this list to other clinically significant genes, such as HLA-DPA1 and HLA-DPB1.

## Supplementary Material

btag231_Supplementary_Data

## Data Availability

HPRC HiFi reads and assemblies are publicly available from https://humanpangenome.org/hprc-data-release-2/ and the list of 233 samples are included as Supplementary File. Nanopore R10 reads include three non-cancer samples (H1437, H2009 and HCC1937) from CASTLE panel (https://github.com/CASTLE-Panel/castle) and HG002 from GIAB (https://42basepairs.com/browse/s3/ont-open-data/giab_2023.05/analysis/hg002/).
