# Supplementary figures and images for: FuFiHLA: a tool for full-field HLA typing from long-read data
Source: Bioinformatics. 2026 May 5;42(5):btag231. doi: 10.1093/bioinformatics/btag231 (PMC13221240; doi:10.1093/bioinformatics/btag231)

HLA-A

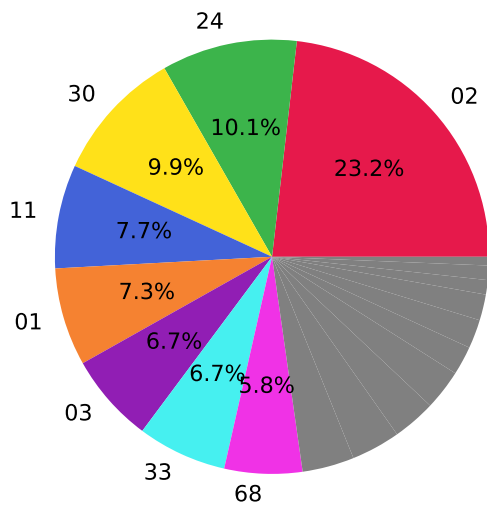

HLA-B

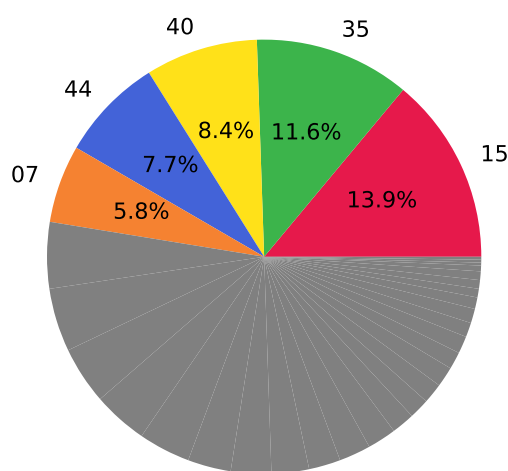

HLA-C

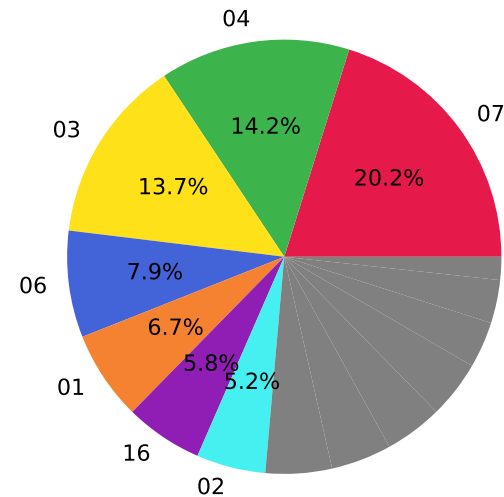

HLA-DRB1

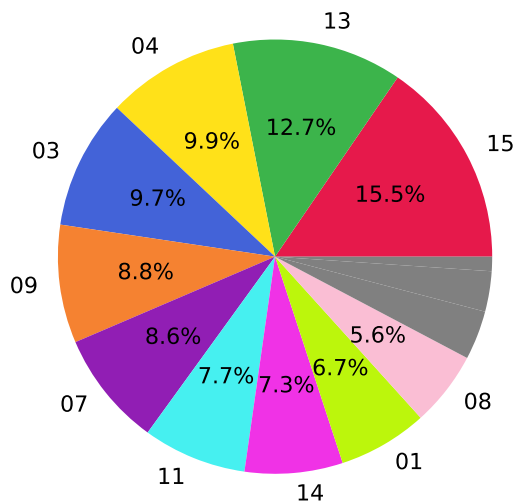

HLA-DQA1

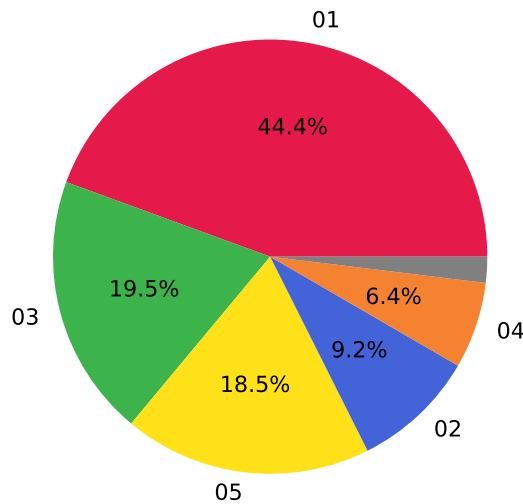

HLA-DQB1

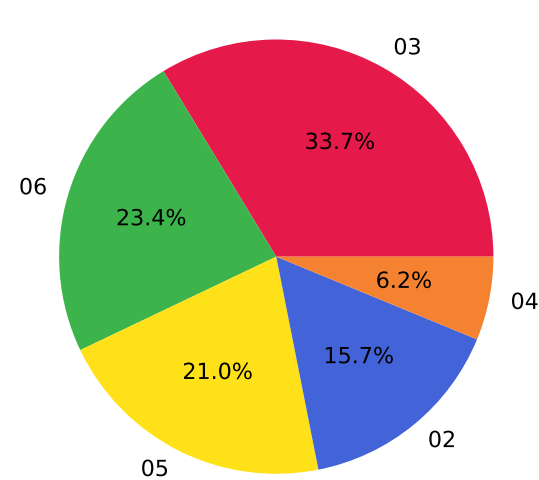

Supplement: btag231_Supplementary_Data [file btag231_supplementary_data.zip › Figure-S1_allele_distribution.pdf]

**EAS(n = 51)**

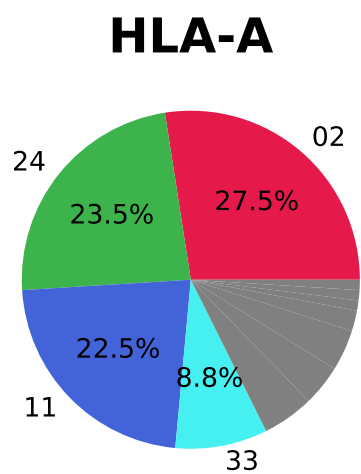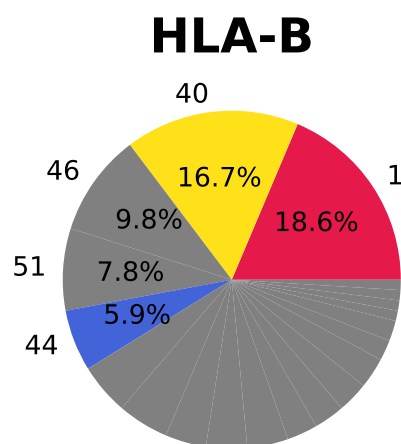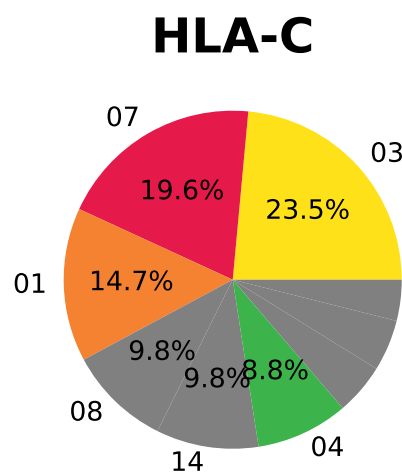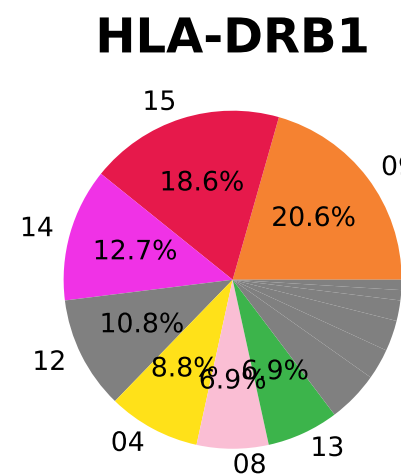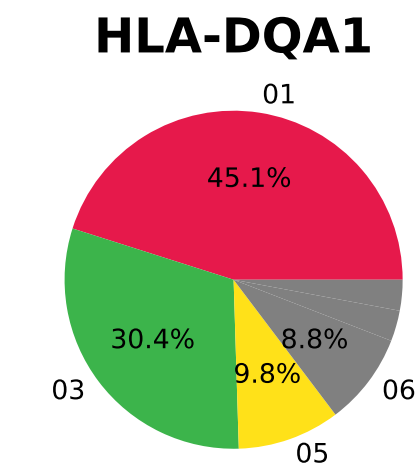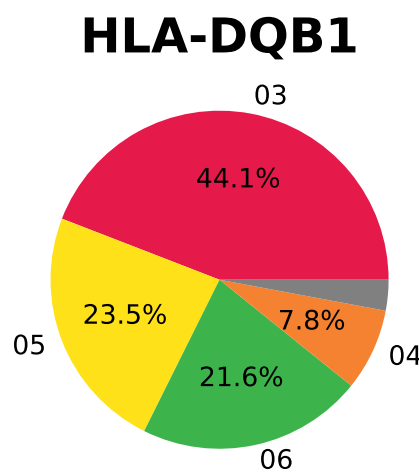

**SAS(n = 37)**

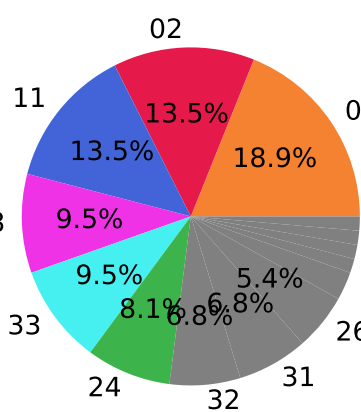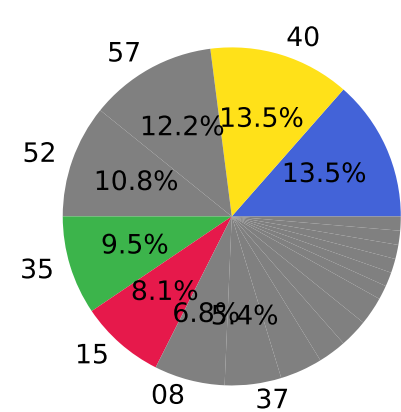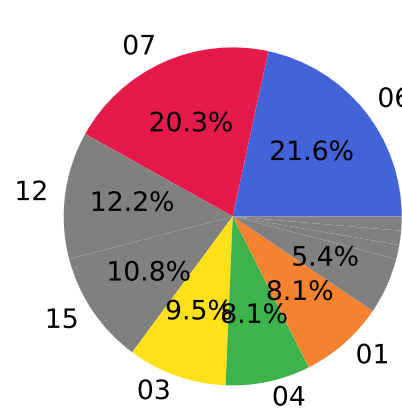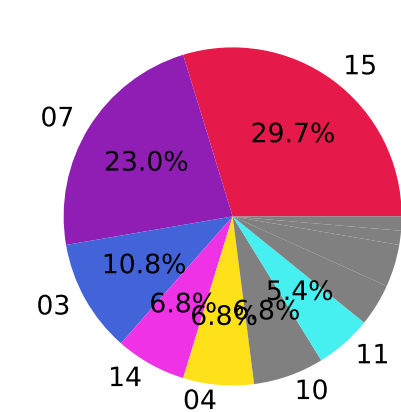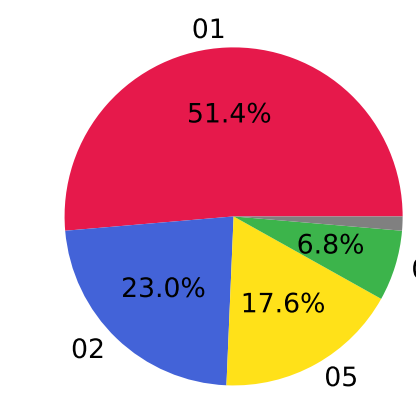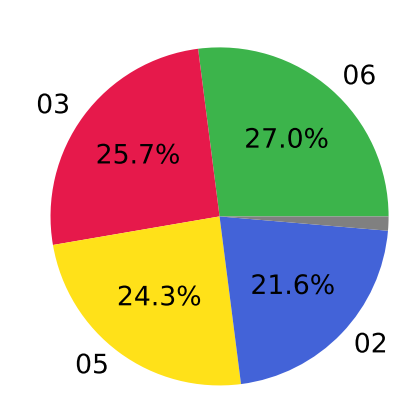

**EUR(n = 31)**

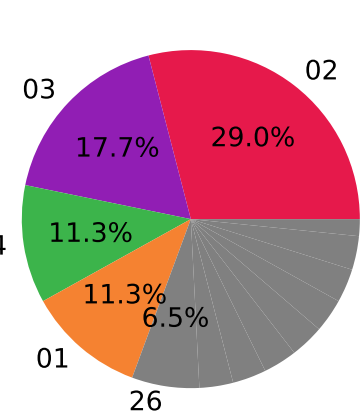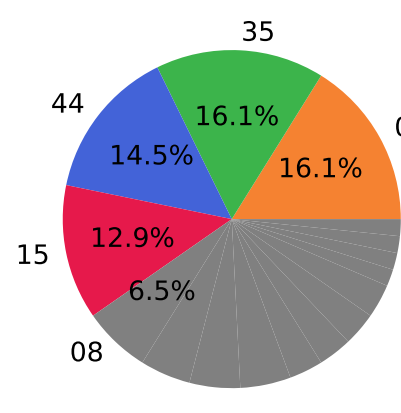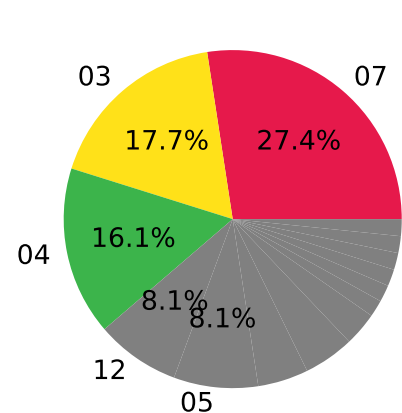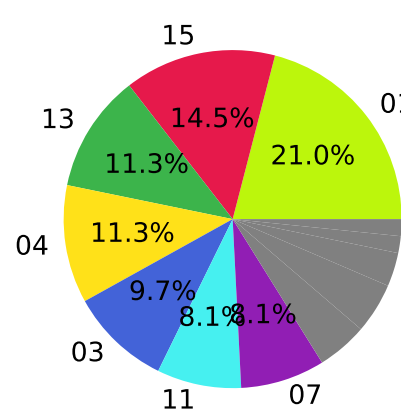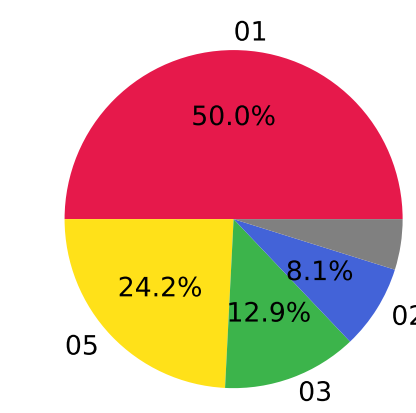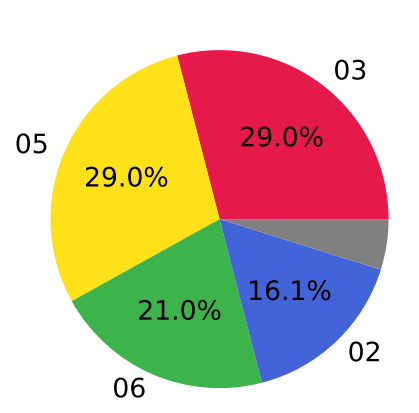

**AMR(n = 45)**

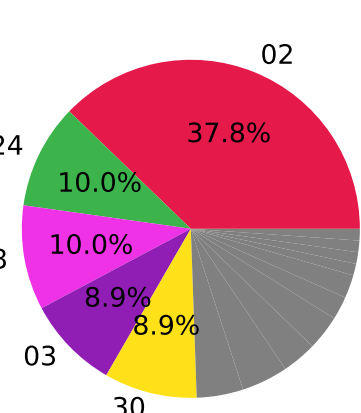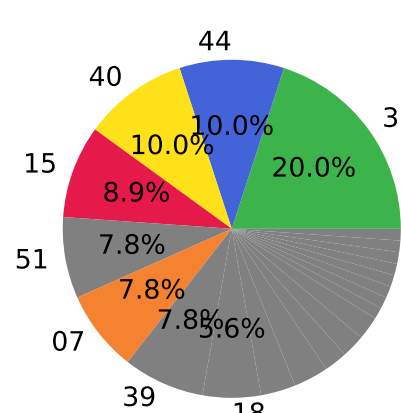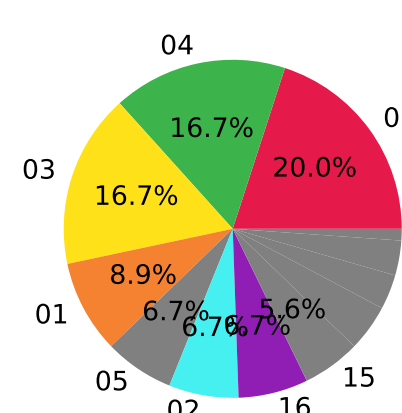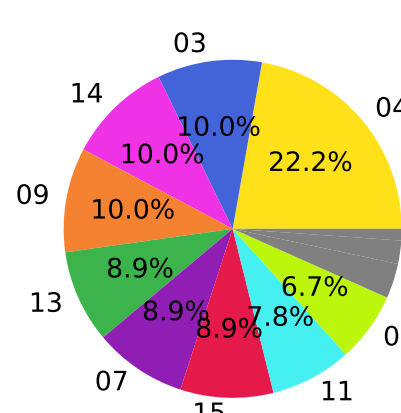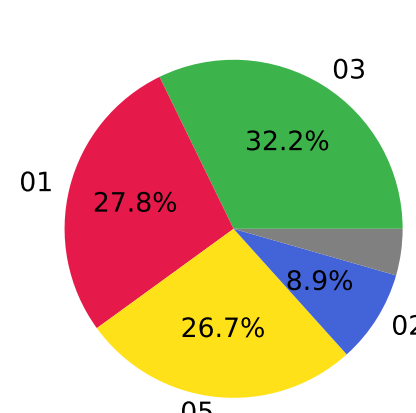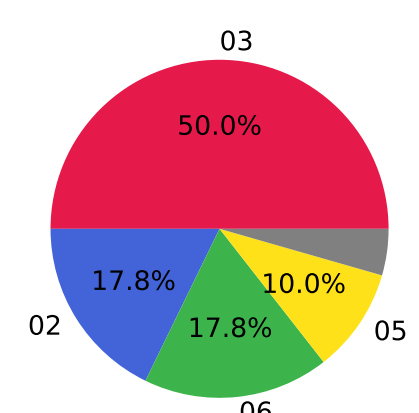

**AFR(n = 69)**

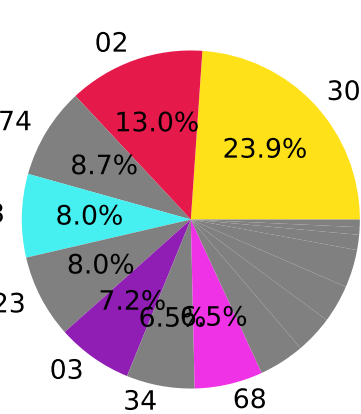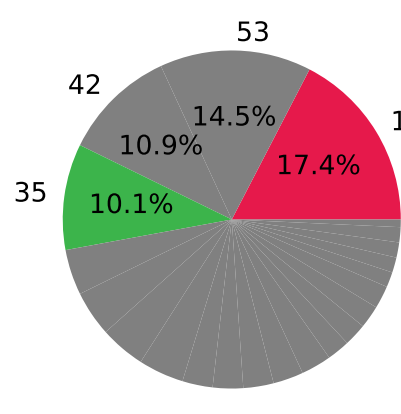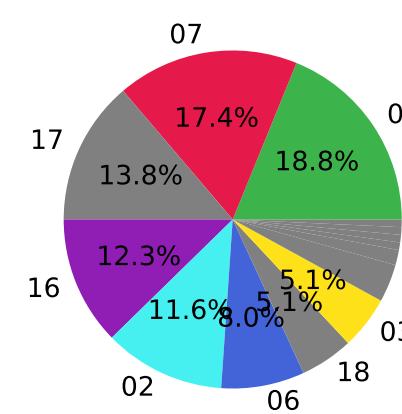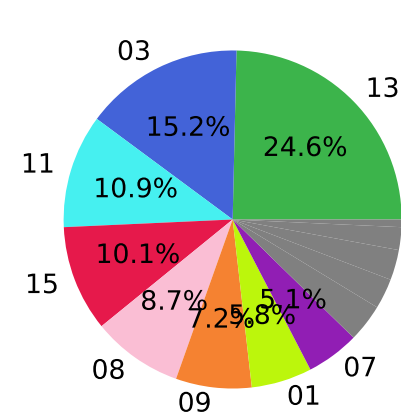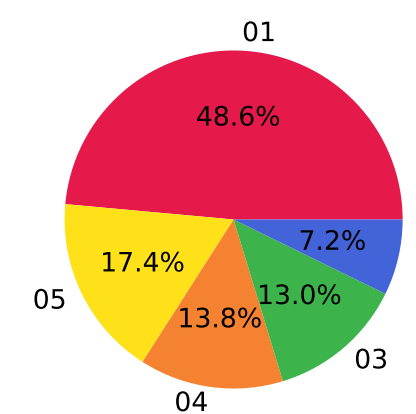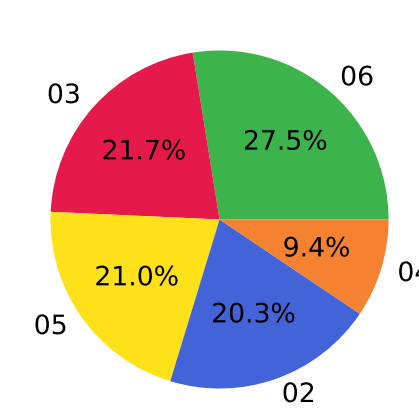

Supplement: btag231_Supplementary_Data [file btag231_supplementary_data.zip › Figure-S2_ANC_specific_allele_distribution.pdf]
